# Disturbance in human gut microbiota networks by parasites and its implications in the incidence of depression

**DOI:** 10.1038/s41598-020-60562-w

**Published:** 2020-02-28

**Authors:** Elvia Ramírez-Carrillo, Osiris Gaona, Javier Nieto, Andrés Sánchez-Quinto, Daniel Cerqueda-García, Luisa I. Falcón, Olga A. Rojas-Ramos, Isaac González-Santoyo

**Affiliations:** 1 NeuroEcology Lab, Faculty of Psychology, National Autonomous University of Mexico (UNAM), Mexico City, México; 2 Laboratorio de Ecología Bacteriana, Instituto de Ecología, UNAM, México, 04510 México; 3 Laboratory of Learning and Adaptation, Faculty of Psychology, UNAM, Mexico City, México; 4 Consorcio de Investigación del Golfo de México (CIGoM), Centro de Investigación y de Estudios Avanzados del Instituto Politécnico Nacional, Unidad Mérida, Departamento de Recursos del Mar, Mérida, Yucatán México; 5 Present Address: UNAM, Parque Científico y Tecnológico de Yucatán, México, 97302 México

**Keywords:** Machine learning, Phylogeny, Microbial ecology, Bacterial host response, Depression

## Abstract

If you think you are in control of your behavior, think again. Evidence suggests that behavioral modifications, as development and persistence of depression, maybe the consequence of a complex network of communication between macro and micro-organisms capable of modifying the physiological axis of the host. Some parasites cause significant nutritional deficiencies for the host and impair the effectiveness of cognitive processes such as memory, teaching or non-verbal intelligence. Bacterial communities mediate the establishment of parasites and vice versa but this complexity approach remains little explored. We study the gut microbiota-parasite interactions using novel techniques of network analysis using data of individuals from two indigenous communities in Guerrero, Mexico. Our results suggest that *Ascaris lumbricoides* induce a gut microbiota perturbation affecting its network properties and also subnetworks of key species related to depression, translating in a loss of emergence. Studying these network properties changes is particularly important because recent research has shown that human health is characterized by a dynamic trade-off between emergence and self-organization, called criticality. Emergence allows the systems to generate novel information meanwhile self-organization is related to the system’s order and structure. In this way, the loss of emergence means a depart from criticality and ultimately loss of health.

## Introduction

It is well documented that parasites can modulate several host’s behavioral patterns^1^, such as feeding, or reproductive behavior^2^. These changes are mediated by physiological mechanisms that include hormonal^3^, immunological^4^ and neurological components^5^. Nevertheless, these physiological levels may also be regulated by other microorganisms like bacterial microbiota that coexist in the same host’s internal environment^6^. In this sense, the host’s behavioral changes might be viewed as the result of the complex communication network between macro and microorganisms that have the ability to modify the mentioned host’s physiological axis^6^. Moreover, the presence of certain bacterial communities should also impact the establishment of parasites and vice versa, parasites could be modifying the bacterial microbiota composition. This bidirectional relation is plausible if both groups compete for similar host’s resources, such as a specific nutrient or an ecological niche, or because of the activation of the host’s immune response due to the presence of parasites, disrupting different homeostatic relations established between bacterial microbiota and its host^6^.

In humans, *Ascaris lumbricoides* a soil-transmitted helminth (STH) that affects more than a third of the world’s population, mainly in low-income populations in developing regions of Africa, Asia, and the Americas^7^. Its infection causes important nutritional deficits for the host^8^, and empirical evidence points out that it impairs the efficiency of cognitive processes, such as memory, learning or even non-verbal intelligence^9^. In particular, the bacteria gut microbiota is the most diverse community of microorganisms with 1183 to 3180 genus reported so far^10^, and is undoubtedly essential to maintain the host health. For instance, to date, at least 50 human pathologies have been associated with changes in the abundance and composition of gut microbiota^11^. Human gut microbiota not only participates in most complex metabolic processes, such as fiber or starch catabolism but also in the protection from pathogens^12–15^. On the other hand, in recent years it has also become evident that gut microbiota creates a two-way communication with the Central Nervous System (CNS). Due to this communication, our gut microbiota composition may be affected by emotional variables such as stress or depression, or changes in the intestinal microbiota may affect motivation and other higher cognitive functions^16,17^.

Advances in sequencing technology have made possible to explore the role of the gut microbiota in a wide range of neurological and psychiatric disorders, including a larger-scale analysis of self-reported conditions as performed by Valles-Colomer and her colleagues^18^ who investigate the gut microbiota compositional covariation with quality of life (QoL) indicators and general practitioner-reported depression in the Belgian Flemish Gut Flora Project (population cohort; n = 1,054). They found that *Coprococcus* and *Dialister* genera were depleted in people with important indicators of depression. Interestingly, although gut microbiota may be affected by several socio-economic and cultural contexts such as differences in food intake^19–21^; or medical practices like the over-ingestion of antibiotics^22^, the gut microbiota ecosystems may also be modified by the interaction with other microorganisms, as is occurring with helminth infections like *A. lumbricoides*^23^. This might be a novel research field, a multi-ecosystemic perspective of the microbiota-gut-brain axis, which remains little explored.

To our knowledge, although no direct relationship has been reported between *Coprococcus* and *Dialister* genera and the helminth *A. lumbricoides*, recent work by Krogsgaard and co-workers^24^ reported that bacteria species strongly associated with irritable bowel syndrome (including *Coprococcus* and *Dialister*) were found at higher abundance in parasite-negative samples compared with parasite-positive samples, with no specific parasite reported.

In addition, although different people even in the same population, may present considerable microbial species variability, there has been recognized that gut microbiota exhibits some sort of ecological stability that translates into the fact that key species tend to remain present for long periods of time^15,25^. This stability property of gut microbiota is considered key for host health and well-being because it ensures that beneficial symbionts and their associated functions are maintained over time^26^.

In that sense, healthy hosts should have gut microbiota in what has been called criticality, the balance between robustness and adaptability^27^. For instance, a healthy microbiota should have sufficient adaptation to respond to external variability, like changes in types of food available; but it also needs to be robust in terms of key bacteria populations. The Criticality Hypothesis, states that systems in a dynamic regime shifting between order and disorder, attain the highest level of computational capabilities and achieve an optimal trade-off between robustness and adaptability^28^. In this framework robustness is associated with order and self-organization, meanwhile, adaptability is related to disorder and information emergence, as we will discuss below. Empirical evidence has related human health to heart, and brain criticality^29–32^, and loss of criticality (mainly by loss of adaptability) with chronic diseases (such as obesity or diabetes) and elderly process^33^. In their work, Huitzil and co-workers (2018)^27^ claim that microbiome and genome networks are critical networks which means that their dynamical behavior is at the brink of a phase transition between order and chaos^34,35^. This idea is supported by the facts that dynamical criticality confers the system properties such as evolvability (i.e., the coexistence of robustness and adaptability)^36,37^, faster information storage, processing, and transfer^38,39^, and collective response to external stimuli without saturation^40^; and in fact, there is solid evidence indicating that gene regulatory networks of real organisms are dynamically critical or close to criticality^41–44^.

This kind of multi-ecosystemic, complex system perspective presents some serious challenges since microbiota contains many diverse species interacting with one another^26^, which makes the full system complex and challenging to understand. Network analysis has proven to be a valuable framework to understand large and complex interacting communities^45^. For instance, network analysis allows studying not only the whole ecosystem but also to focus on key bacteria for microbiota-gut-brain axis subnetworks (communities). In particular, Valles-Colomer and co-workers^18^ have reported specific gut bacteria genus related to wellbeing and depression. Therefore, in the present work at first, we explore the gut microbiota ecosystem considering whether the presence of the STH *A. lumbricoides* predicts the gut microbiota network for adults and children (both female and male) in two poor indigenous non-industrialized communities with the highest index of STH infections in México. Secondly, we focus on how *A. lumbricoides* infection is associated with subnetworks of bacteria communities strongly related to human depression symptomatology: *Coprococcus* and *Dialister* with a negative relation.

## Results

**Figure 1:**
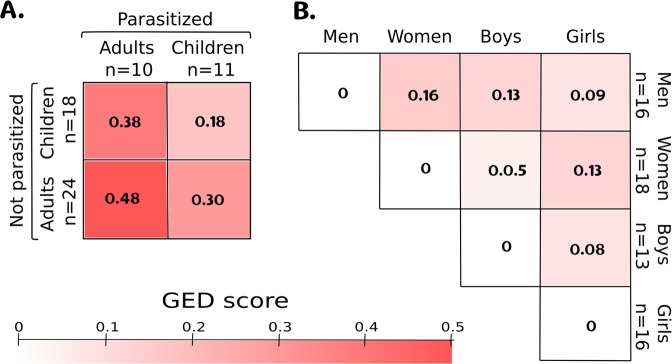
This figure show Graph Edit Distance (GED) comparing different pairs of networks. The scale [0-1] goes from no difference to a total difference. (**A**) Show the comparisons between Not Parasitized (NP) Vs Parasitized (P) populations divided by Adults and Children. (**B**) Differences between networks disaggregated by age and gender. The network of the microbiota in Adults is more affected by the presence of parasites showing a 48% difference between treatments. Children are the least affected, differing only by 18%. In all cases, the presence of parasites showed more differences when comparing the magnitudes by treatments than between ages or sexes.

We present the analysis of The Graph Edit Distance (GED) analysis, which is a tool used for comparing complete networks structures. In Fig. 1. A we show GED scores to compare Not Parasitized (NP) vs Parasitized (P) populations divided by age group: Adults and Children. For NP we analyzed 24 adults and 18 children; meanwhile, for P there were 10 adults and 11 children. The scale goes from no difference (0) to different (1). Major differences are observed between Adults-NP Vs Adults-P that differ around 48%, followed by Children-NP Vs Adults-P that differ some 38% and finally Adults-P vs Children NP with a difference of 30%. The smallest differences (less affection from parasitosis) were found between Children-NP Vs Children-P. On the other hand, Adults showed greater affectation due to the presence of parasites. In order to compare the magnitude of differences, in Fig. 1B we show the GED scores paired, disaggregating data using age and gender (16 men, 18 women, 13 boys, and 16 girls). In all cases, the magnitude of the difference is less than that caused by the presence of parasites.

**Figure 2:**
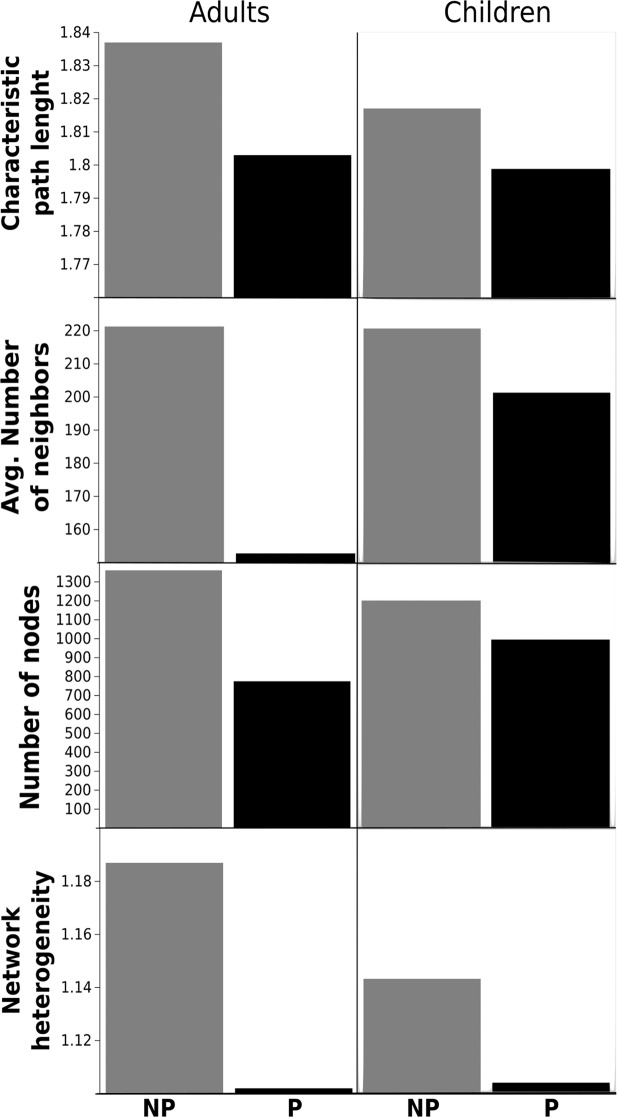
Standard network analysis measurements. In all the network measurements of individuals parasitized both Adults and Children shown lower Characteristic Path Length, Average number of neighbors, Number of nodes and Network heterogeneity. Gray bars represent No Parasitized individuals while Black bars represent Parasitized individuals.

**Figure 3:**
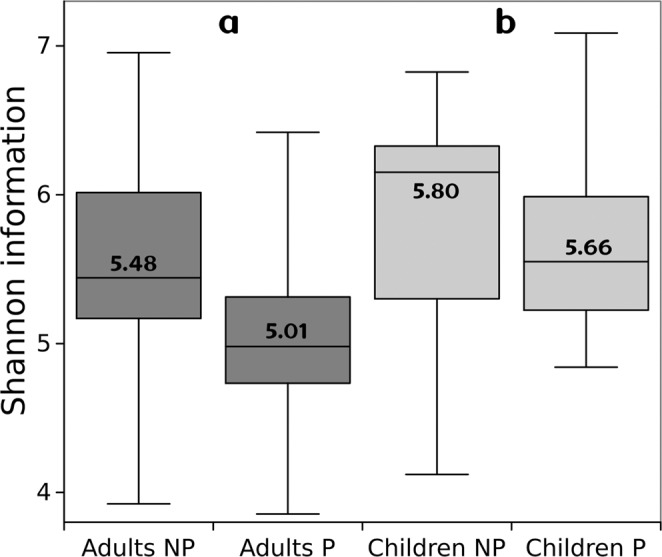
This figure show a box-plot with median and quarterlies of Shannon information as a measurement of information emergence and its mean in number. We found that Emergence is bigger in children than in adults (t = 2.36, p = 0.02) and as was discussed this may implies more adaptability. On the other hand, adults show a diminish in Shannon Information (S), due to the presence of parasites (t = 1.76, p = 0.04).

Parasitosis also altered other standard network analysis measurements including Characteristic Path Length, Average number of neighbors, Number of nodes and Network heterogeneity (Fig. 2). Parasitized Adult and Children networks show a decrease in all measures, which are related with different aspects of complexity that in turn may be defined^46^ as the product of emergence (adaptability), measured directed as Shannon Information (S), and self-organization (robustness) measured as its complement. Hence Complexity (C) may be calculated by the equation

1 $$C=S(1-S)=(S-{S}^{2}),$$

which is a quadratic form of information emergence meaning too little or too much emergence implies lower levels of complexity. Following the above, we calculated the S networks shown in Fig. 3, which may give us information about the emergence of systems. We found that Emergence is bigger in children than in adults (t = 2.36, p = 0.02) and as was discussed, this implies more adaptability. On the other hand, adults showed a diminish in S, due to the presence of parasites (t = 1.76, p = 0.04).

In turn, Bak and Paczuski^47^ has pointed out that Complexity arises from the tendency of large dynamic systems to become critical. Then, as Complexity and Criticality are inherently connected^48^, a lost in Complexity translates in a depart from Criticality and most likely from healthy states too.

**Figure 4:**
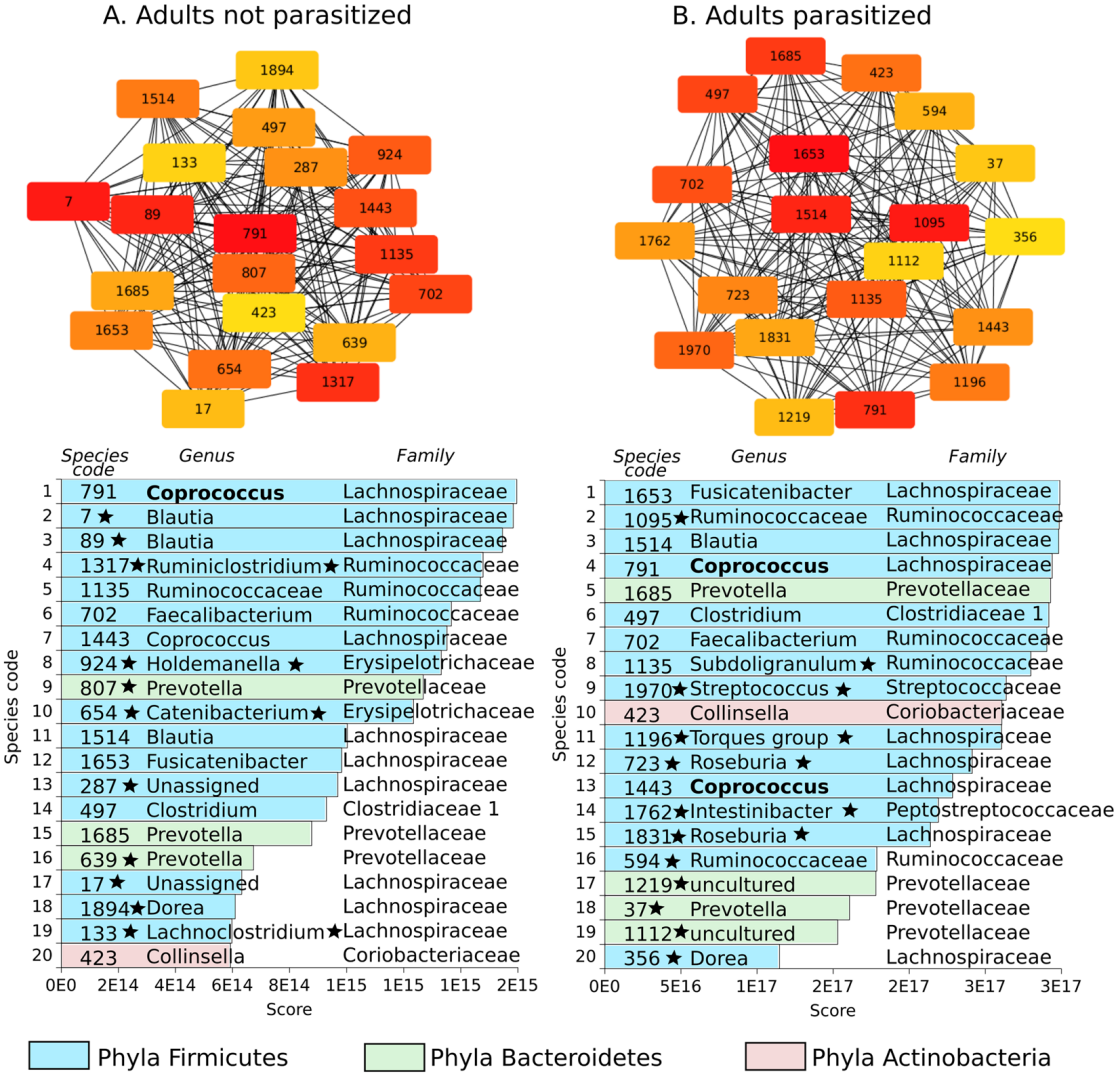
This figure show the 20 most important species for Adults-NP Vs Adults-P, calculated using the Maximal Clique Centrality (MCC) algorithm implemented in cytoHubba package^49^. Inside the boxes are the species identifier number. The list shows each species score along side with genera, family and phyla.Colors encode phyla while star symbol represents which of them are present only in NP or P networks. When star is next to number, it means the species is only present in NP or P; when the star is next to a name it is the same but for genus.

**Figure 5:**
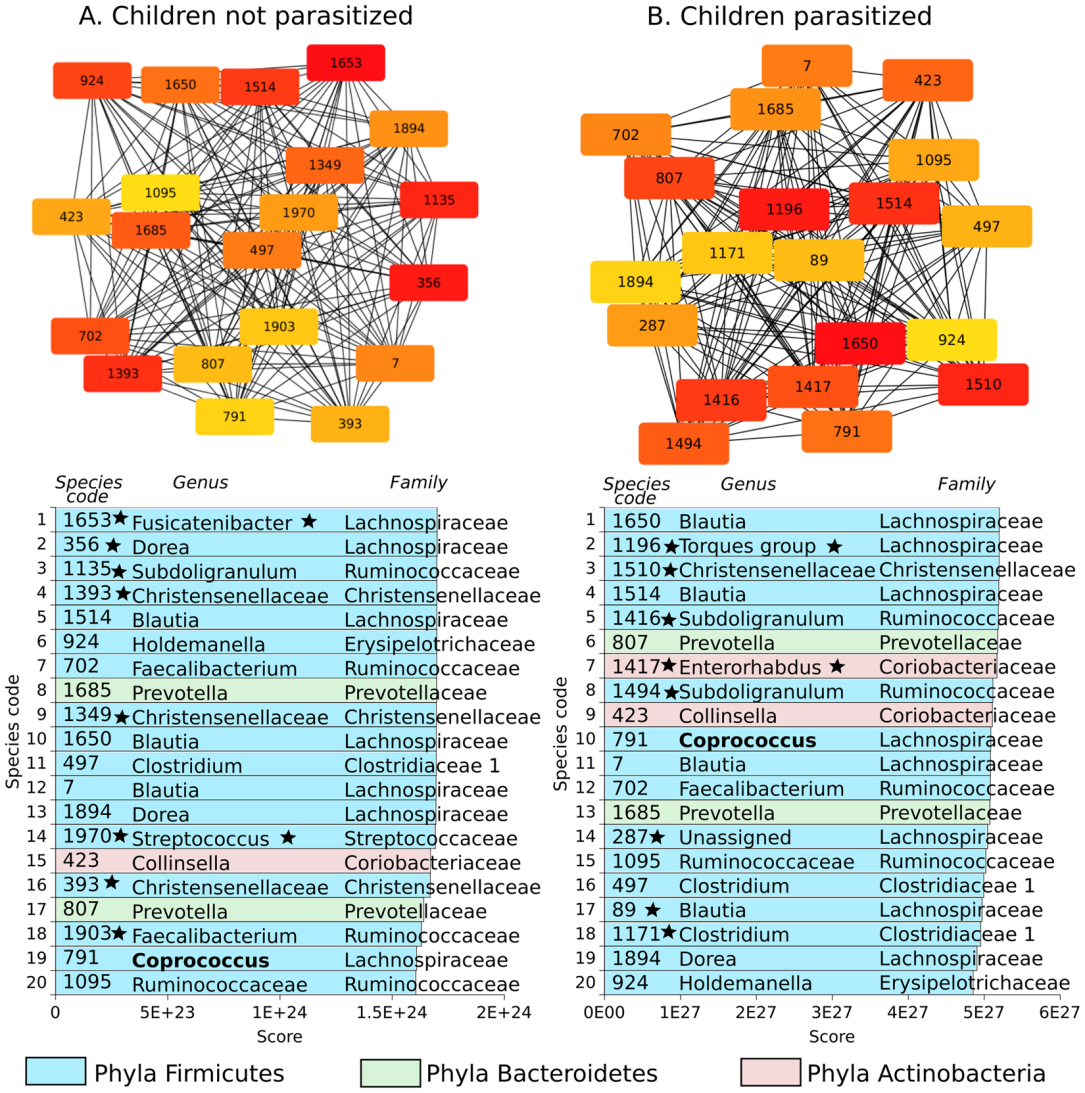
This figure show the 20 most important species for Children-NP Vs Children-P, calculated using the Maximal Clique Centrality (MCC) algorithm implemented in cytoHubba package^49^. Inside the boxes are the species identifier number. The list shows each species score along side with genera, family and phyla. Colors encode phyla while star symbol represents which of them are present only in NP or P networks. When star is next to number, it means the species is only present in NP or P; when the star is next to a name it is the same but for genus.

In order to understand in more depth the difference between P and NP networks we construct specific subnetworks for the most relevant species in terms of weight and connectivity, using the Maximal Clique Centrality (MCC) algorithm implemented in cytoHubba package^49^. Figures 4 and 5 shows the 20 most important species according with MCC. Figure 4 correspond to adults subnetwork and Fig. 5 for children, in both cases with or without parasites. Inside the boxes are the species identifier number, listed below. Colors encode phyla while star symbol represents which of them are present only in NP or P networks. When star is next to number, it means the species is only present in NP or P; when the star is next to a name, its the same but for genus.

We observed that although there are clear differences for complete networks (Figs. 1 and 2), MCC subnetworks did not show substantial changes in the 20 most important species between NP and P. This result makes sense since it has been acknowledged that gut microbiota has some kind of ecological stability which translates into the fact that some species considered as important, tend to stay present after disturbances for a long period^15,25^

**Figure 6:**
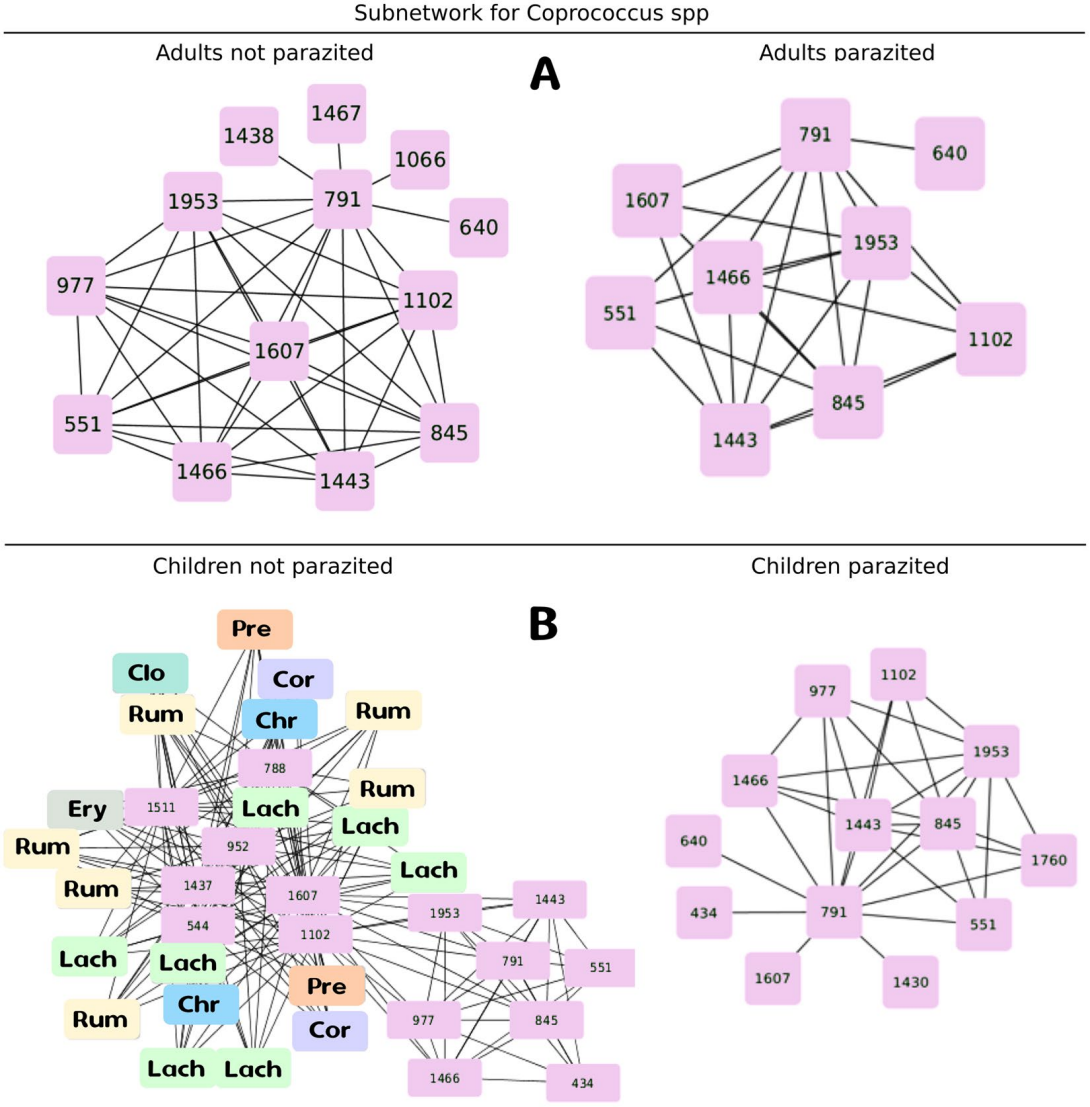
Subnetwork for bacteria species of *Coprococcus* genus, related to the incidence of depressive disorders as reported by (Valles-Colomer *et al*., 2019). Upper subfigure corresponds to Adult population, in the left Not parasitized Adults (NP) and on the right Parasitized Adults (P). Lower subfigure corresponds to Children populations, in the left Not parasitized Children (NP) and on the right Parasitized Children(P). Pink boxes are the species code for the species of the genus *Coprococcus* founded in the subnetwork. In color boxes there are the species with which they interact in the subnetwork. The letter code inside each box indicates the families: Christensenellaceae (Chr), Clostridiaceae (Clo), Coriobacteriaceae (Cor), Erysipelatrichaceae (Ery), Lachnospiraceae (Lach), Peptostreptococcaceae (Pep), Prevotellaceae (Pre), Ruminococcaceae (Rum).

**Figure 7:**
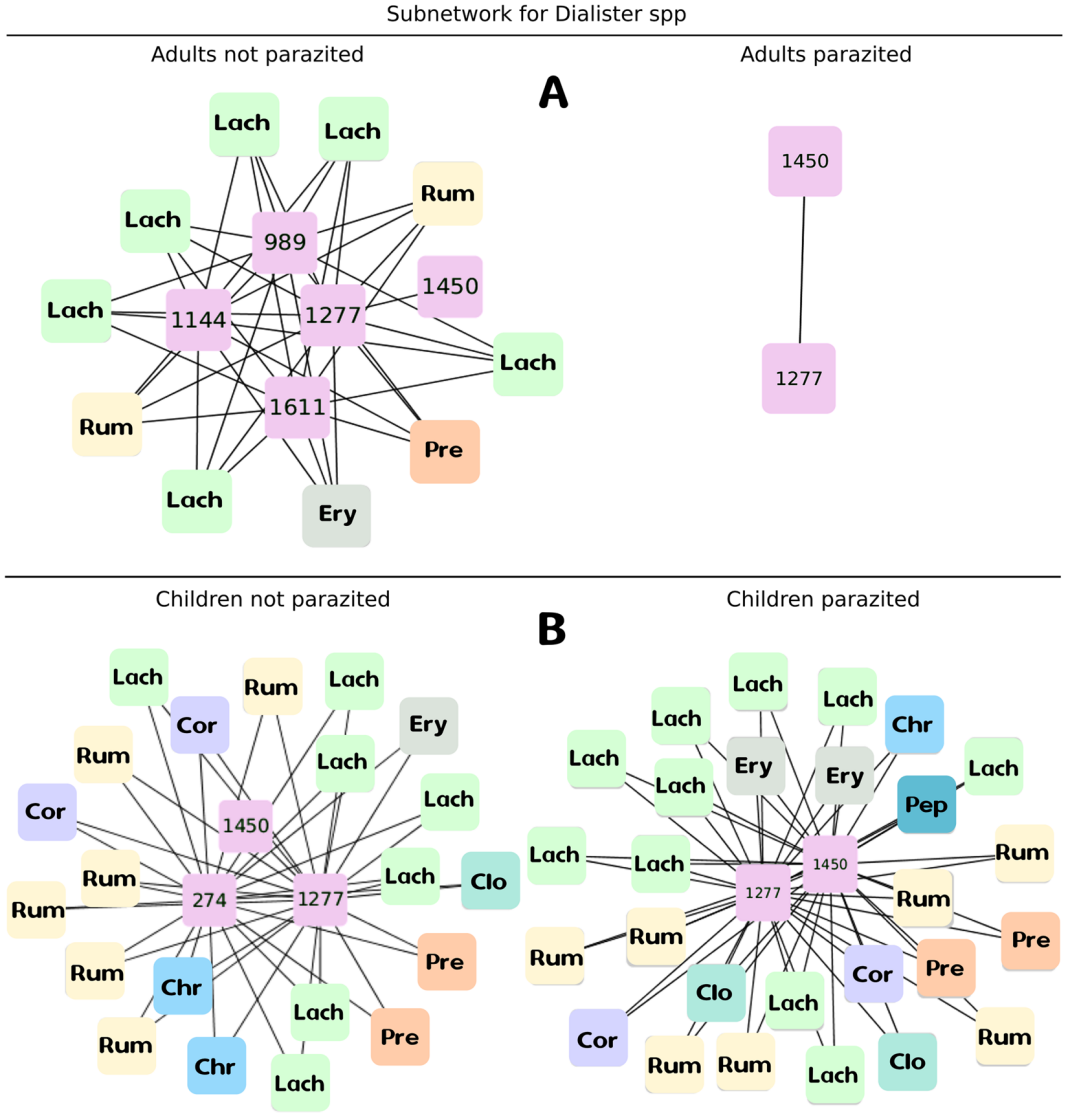
Subnetwork for bacterial species of *Dialister* genera, related to the incidence of depressive disorders as reported by (Valles-Colomer *et al*., 2019). Upper subfigure corresponds to Adult population, in the left Not parasitized Adults (NP) and on the right Parasitized Adults (P). Lower subfigure corresponds to Children populations, in the left Not parasitized Children (NP) and on the right Parasitized Children(P). Pink boxes are the species code for the species of the genus *Dialister* found in the subnetwork. In color boxes there are the species with which they interact in the subnetwork. The letter code inside each box indicates the families with the same code of the previous figure.

To see the effect of the parasites on the specific subnetworks of genera of bacteria *Coprococcus* and *Dialister*. We compared the subnetworks of both genera for Adults and Children Parasitized and Non-Parasitized. A linear model was carried out to explain the variation of the wealth concerning the presence of parasites, age and species (${R}^{2}$ = 0.78, p = 0.001) and a marginally significant negative relationship was found (t = $-1.97$, p = 0.05) between wealth and the presence of parasites. But beyond that number of nodes (species) decreases with the presence of parasites, the results of subnetworks shown in Figs. 6 and 7 allow us to observe how interactions with other species within the network affected. For *Coprococcus*, children present more richness of species than adults and in both cases, parasitosis reduces the number of species. Besides, Coprococcus subnetwork in NP-Adults did not show any link with other families of bacteria which was maintained in P-Adults, while in Children the presence of *A. lumbricoides* resulted in lost of interactions of Coprococcus with other families of bacteria. On the other hand, subnetworks of the *Dialister* genus are very interesting because NP-Adults subnetworks shown greater richness of species but fewer interactions with other families of bacteria than children. And in this case, the presence of the parasite in Adults resulted in a network collapse with only two species without interaction. In contrast, although the number of species in Children was also reduced, interactions increased even with other new species, such as the uncultured bacterium of Intestinibacter genus of Peptostreptococcaceae family.

## Discussion

We have studied from a complex systems perspective the effect of the STH *A. lumbricoides* in the network properties of the host’s gut microbiota, focusing on particular effects of the disturbance on key bacterial genera in which their absence is strongly related with depression: *Coprococcus* and *Dialister*. We found that the presence of the parasite *A. lumbricoides* induces a loss of the microbiota network features related with its complexity, such as path length, heterogeneity, number of nodes and neighbors. We also found that for the genera *Coprococcus* and *Dialister* there was a loss in information emergence and then depart from criticality, which has been identified as a fingerprint of human health.

The interaction of parasites with microbiota is an open hot field of study^18^ with complex interplays that reflects into both quality of life and depression. In the case of adults and children from the indigenous communities studied, we observed that the presence of *A. l umbricoides* alters the structure of the gut microbiota networks, being more affected in adults who change by 48% in presence of the intestinal parasite. Moreover, adults have the least emergency (adaptability) and it decreases significantly when presenting *A. lumbricoides*. On the other hand, the population of children that had initially greater diversity turns out to be more resistant and less affected to disturbances in the form of parasitism. An interesting question is whether our microbiota is losing criticality (via loss of adaptability) as we grow, as has been observed in the electrical activity of the heart^33^.

Interestingly, although the presence of *A. lumbricoides* decreases the total nodes (species) over the complete networks, if the analyses is done over the 20 most relevant species (according to the MCC) sub-networks some changes arise, especially at genus levels. This may indicate that there is a certain “kernel” of species that are maintained despite the disturbance giving stability to the microbiota system^15,25^.

The novelty of these analyses is that they allow us to analyze the interactions from a complex perspective, allowing us to see the whole system but also how different components are affected. In this sense, when analyzing the sub-networks for species related to depression, we observed that although in general species are lost, the interactions between them provide us with new insights. For example, on the one hand, higher quality of life indicators were related to Butyrate-producing species as *Coprococcus* sp. presence. On the other hand both *Dialister* and *Coprococcus* genera were diminish under depression condition, even when antidepressants confounding effects were taking into account^18^.

We show that the presence of *A. lumbricoides* impacts in a particular way the subnetwork for these bacteria genera, first reducing the number of species that compose each genera in the net, and secondly reducing the interactions of these with other species. So, we can have different second-order effects by affecting interactions with other species. How the interactions affect the species is complex and we still need to know a lot of particularity, but it could mean that the presence of parasites can promote relevant changes in networks of bacterial communities strongly related with the incidence of depression. This makes sense, from a Criticality Hypothesis standpoint, with lower values of network analysis measurements and Shannon Information for parasitized Adults and Children compared with Non-Parasitized individuals. These hypotheses pose that when a system reach an optimal balance between adaptability and robustness, the system is in criticality related with the highest level of computational capabilities. In this context, emergence can be measured under particular set of parameters for continuous distributions, as we did in this work by Shannon information^50^.

Recent results show convincing evidence that human health requires that systemic physiological signals, such as heart rate, be at criticality^29–32,51^. Our results show that due to the presence of parasites, there is a depart from criticality via a diminish of emergence (adaptability) and then a loss of health. Nevertheless, the net effect of parasites interacting with microbiota maybe not as straightforward as some recent studies suggest^26^, since we have been co-evolving with them and some types and intensity of parasitism might impose some sort of stressor for the microbiota, which may produce a hormesis effect contributing to healthier states. This second order effect relates to Taleb’s ideas^52^ about antifragility in medicine. Antifragility is a property that enhances the capability of a system to respond to external stressors in a nonlinear convex manner in the payoff space. Antifragile systems take advantage of volatility and stressors, so in their absence, this property could be lost. Moreover, in^53^ the authors show how an antifragile system is at or sufficiently near to criticality.

In this work, we have shown the importance of studying second-order effects of microbiota interaction with parasites, for example in terms of potential effects over depression processes. It is also clear that a complexity approach using network and information theory has great potential in the field. In this sense, we propose a promising line of research in terms of how microbiota respond to disturbances using the ideas of antifragility.

Finally, we consider that some future research directions in this area may be to analyze this gut-microbiota-brain axis with brain imaging techniques such as quantitative electroencephalography. This could allow us to evaluate how brain connectivity could be associated with particular gut microbiota networks, and how a disturbance episode in the microbiota could in turn, impacts this brain connectivity. With this kind of ecosystemic approach it would be also important go beyond composition and structure of the microbiota, incorporating functions, and in the same way it would be also very interesting to deepen into ecosystem antifragility^54^ of the microbiota ecosystem.

## Methods

### Study site

Mexico has at least 58 native and independent indigenous groups^55^, whose lifestyle practices strongly differ from the typical lifestyle present in “Western, Educated, Industrialized, Rich and Democratic” (W.E.I.R.D.) populations^56^. In particular, the Me’Phaa people, from the southeast region of México known as the “Montaña Alta” of the state of Guerrero, is one of the most contrasting groups^57^. In these communities, there is almost no access to allopathic medications, and there is no health service, plumbing, or system of water purification. Water for washing and drinking is obtained from small wells^58^. Therefore, these communities represent the lowest income in the country, the highest index of child and adult morbidity and mortality by intestinal infection (children’s age from 0 to 8 years old^58,59^, which is the highest vulnerability and death risk age^60^), and the lowest access to health services. These conditions were determined by last 10 years of statistical information obtained from National information system of access to health^59^. Most Me’Phaa speak only their native language, and the closest large town (the main municipal town) is two hours away by dirty-road. Our data were collected from two of these indigenous communities; Plan de Gatica (17.130000${}^{\circ }$, $-99.12111{1}^{\circ }$, EASL: 510 m) and el Naranjo (17.135556${}^{\circ }$, $-99.04611{1}^{\circ }$, EASL: 860 m) . Distance between these two communities is about 30 km^61^, and their socio-economic and cultural patterns are the same between them^61^. Although allopathic medication is practically absent in these communities^59^, we selected only participants that have not taken any allopathic medications during the last two years prior to study, such as antibiotics or anthelmintic treatment. Samples were taken from 63 individuals in total- 35 from Plan de Gatica and 28 from El Naranjo. Children were aged 5 to 10 years old, and adults were between 18 and 45. We sampled 29 children in total: 16 (7 from Gatica and 9 from Naranjo) and 13 (8 from Gatica and 5 from Naranjo), whose average age was 7.6 +/$-1.8$ years. Among adults, we sampled 34 total. 18 were women (10 form Gatica and 8 from Naranjo) and 16 were men (10 from Gatica and 6 from Naranjo). The average age was 30.48 +/$-$7.79 years.

### DNA extraction from feces

Characterization of composition and abundance of the participants’ intestinal microbiota was done by a non-culture method implementing a High throughput strategy with 16S ribosomal amplicons and mass sequencing, using illumina platform. Therefore, two grams of fecal samples from each participant were received in sterile containers, then transferred to 1.5 milliliter Eppendorf tubes using sterile technique, and transported in liquid nitrogen at negative 80 degrees Celsius until DNA extraction. Metagenomic fecal DNA was extracted using the DNeasy Blood & Tissue kit (Qiagen, Valencia, CA) according to the manufacturer’s protocol. Briefly, once feces were collected into 1.5mL sterile tubes, we diluted each sample with 180 $\mu$l of ATL extraction buffer with 20 $\mu$l proteinase K (10 mg ml${}^{-1}$). Then, we mixed Tubes thoroughly by vortexing and incubated at 56 ${}^{\circ }$C at 1500 rpm for 50 min. 200 $\mu$l of AL Buffer with 200 $\mu$l ethanol (96–100%) were added and mixed thoroughly by vortexing. The mixture obtained was transferred into the DNeasy Mini spin column, washed with Buffer AW1 and then with AW2. Finally, the DNA was eluted with 200 $\mu$l of AE Buffer and precipitated with absolute ethanol, 0.1 volume 3 M sodium acetate and 2 $\mu$l glycoblue. DNA was resuspended in 30 $\mu$l of molecular grade water and stored at $-{20}^{\circ }$C until PCR amplification.

### Amplification and sequencing of the 16S rRNA gene

In order to obtain the 16S rRNA gene sequencing of the participant’s DNA samples, we performed a PCR-amplified using the hypervariable V4 region of this gene with universal bacteria/archaeal primers 515F/806R following the procedures reported by Caporaso *et al*.^62^, Carrillo *et al*.^63^ and Osiris-Gaona*et al*.^64^. PCR reactions (25 $\mu$l) contained 2–6 ng of total DNA, 2.5 $\mu$l Takara ExTaq PCR buffer 10X, 2 $\mu$l Takara dNTP mix (2.5 mM), 0.7 $\mu$l bovine serum albumin (BSA, 20 mg ml${}^{-1}$), 1 $\mu$l primers (10 $\mu$M), 0.125 $\mu$l Takara Ex Taq DNA Polymerase (5 U $\mu$l-1) (TaKaRa, Shiga, Japan) and nuclease-free water. All samples were amplified in triplicate implementing a PCR protocol that includes an initial denaturation step at 95 ${}^{\circ }$C (3 min), 35 cycles of 95 ${}^{\circ }$C (30 s), 52 ${}^{\circ }$C (40 s), 72 ${}^{\circ }$C (90 s), and a final extension (72 ${}^{\circ }$C, 12 min). The triplicates were pooled and purified using the SPRI magnetic bead, AgencourtAMPure XP PCR purification system (Beckman Coulter, Brea, CA, USA), and the 16S rRNA fragments ($\sim$20 ng per sample) were sequenced on an IlluminaMiSeq platform (Yale Center for Genome Analysis, CT, USA), generating $\sim$250 bp paired-end reads. All sequence data are available from the NCBI Bioproject number PRJNA593240.

### Analysis of the sequence data

The paired-end 2 $\times$ 250 reads were processed in QIIME2^65^. The reads were denoised with the DADA2 plugin to resolve the amplicon sequence variants (ASVs). Reads at forward- and reverse- were truncated at 200 pb, and chimeric sequences were removed using the “consensus” method. As was implemented by Osiris-Gaona *et al*., 2019, Representative ASV sequences were taxonomically assigned using the “classify- consensus-vsearch pluggin”, using the SILVA 128 database as a reference. An alignment was performed with the MAFFT algorithm^66^. After masking positional conservations and gap filtering, then a phylogeny was built with the FastTree algorithm^67^. Plastidic and mitochondrial ASVs were filtered out, then samples were rarefied to a minimum sequencing effort of 20 000. In order to perform statistical analysis, the table of abundance and phylogeny was exported to the R environment using the phyloseq, vegan and ggplot2 packages^68–70^.

Plastidic ASVs were filtered out of the samples (for subsequent separate analysis), then the samples were rarefied to a minimum sequencing effort of 10 000. Counts of plastidic ASVs (filtered before rarefaction) were normalized with the cumulative sum scaling (CSS) method with the metagenomeSeq package^71^. Both, Faith’s Phylogenetic Diversity and Shannon’s Diversity Index were used to calculate the alpha or total diversity of the ASVs obtained.

### Determination of *A. lumbricoides* presence

The presence of *A. lumbricoides* was done in the same participant’s fecal samples used to determine the composition and abundance of its microbiota. The identification of this nematode was done through light field microscopy following the protocol of “Mini-FLOTAC”, standardized by Cringoli *et al*.^72^. This “Mini-FLOTAC” protocol is a novel and more sensitive quantitative method for the identification of STH compared to other techniques, such as Kato-Katz^73^.

### Network and subnetworks analyses

From the dataset of ASVs (i.e. bacteria species) relative abundances in fecal samples, we used the Cooccur package (https://cran.r-project.org/web/packages/cooccur/cooccur.pdf) in R^74^ to construct a co-occurrence matrix and use it as weight in networks for: Adults and Children with and without parasites.

Then we used the CytoHubb app inside the open source Network Analysis software CytoScape^75^ for exploring nodes importance in terms of different topological measurements such as Edge Percolated Component (EPC), Maximum Neighborhood Component (MNC) or Degree among others for ranking nodes (species). In particular we retain the 20 most important species using Maximal Clique Centrality (MCC) which has been reported to be the best option for this^49^.

Once ranked we construct sub networks and compare them in terms of species composition. In a recent work in Nature^18^ the authors report specific gut bacteria related with depression, which we look for in our data and construct subnetworks for each one. Beyond a composition analysis, we used an implementation of Graph Edit Distance (GED) a measure of similarity (or dissimilarity) between two graphs in the CytoGEDEVO app^76^. The GEDEVO^77^ is a method for global topological graph alignment that minimizes graph edit distance (GED) by using an evolutionary algorithm to find the optimal alignments using both crossover and random mutation to reach better scoring after each iteration.

We used CytoGEDEVO to calculate the pared distance between fathers, mothers, daughters and sons. And then between children (male and female) with and without parasites; parents (male and female) with and without parasites.

### Ethical compliance

All relevant ethical regulations were included in the study procedures with the previous approbation of the Committee on Research Ethics of the National Autonomous University of México (FPSI/CE/01/2016). In addition, this study runs in accordance with the ethical guidelines of the Official Mexican Law for the health (NOM-012-SSA3-2012). All adult participants signed a written informed consent, and for the case of participants under 18 years, we obtained a specific written informed consent from their parents, or legal guardians according with the normativity of Mexican Law.

## Data Availability

All sequence data are available from the NCBI Bioproject number PRJNA593240, or from the corresponding author on reasonable request.
